# gaftools: a toolkit for analyzing and manipulating pangenome alignments

**DOI:** 10.1093/bioinformatics/btag406

**Published:** 2026-07-29

**Authors:** Samarendra Pani, Fawaz Dabbaghie, Tobias Marschall, Arda Söylev

**Affiliations:** Institute for Medical Biometry and Bioinformatics, Medical Faculty and University Hospital Düsseldorf, Heinrich Heine University Düsseldorf, Düsseldorf 40225, Germany; Center for Digital Medicine, Heinrich Heine University Düsseldorf, Düsseldorf 40225, Germany; Institute for Medical Biometry and Bioinformatics, Medical Faculty and University Hospital Düsseldorf, Heinrich Heine University Düsseldorf, Düsseldorf 40225, Germany; Center for Digital Medicine, Heinrich Heine University Düsseldorf, Düsseldorf 40225, Germany; School of Biological Sciences, Seoul National University, Seoul 08826, Republic of Korea; Institute for Medical Biometry and Bioinformatics, Medical Faculty and University Hospital Düsseldorf, Heinrich Heine University Düsseldorf, Düsseldorf 40225, Germany; Center for Digital Medicine, Heinrich Heine University Düsseldorf, Düsseldorf 40225, Germany; Institute for Medical Biometry and Bioinformatics, Medical Faculty and University Hospital Düsseldorf, Heinrich Heine University Düsseldorf, Düsseldorf 40225, Germany; Center for Digital Medicine, Heinrich Heine University Düsseldorf, Düsseldorf 40225, Germany; Department of Computer Engineering, Necmettin Erbakan University, Konya 42090, Türkiye

## Abstract

**Motivation:**

Linear reference genomes are ubiquitously used in genomics research, despite known biases associated with their use. In recent years, there has been a shift towards graph-based reference genomes to address some of these biases, which has required development of new algorithms and file formats. This has created a necessity for new tools capable of utilizing these formats and performing operations similar to those carried out by traditional methods.

**Results:**

In this paper we present “gaftools,” a multi-purpose tool that introduces several utilities for processing graph alignments in GAF format. gaftools enables users to index and sort alignments, with graph ordering serving as a necessary step for the sorting process. Additionally, it allows users to view subsets of alignments and perform realignment using the wavefront alignment algorithm, among other features. Many of these functionalities are inspired by SAMtools, which provides similar operations for linear genomes, while gaftools adapts and extends them for pangenomes.

**Availability:**

gaftools is available under MIT license at https://github.com/marschall-lab/gaftools.

## 1 Introduction

Linear reference genomes are inadequate to represent the genetic diversity of a species, which leads to several limitations, including population-specific read mapping biases (Garrison *et al.* 2018, Chin *et al.* 2023, Porubsky *et al.* 2023). With the recent efforts of the Human Pangenome Reference Consortium (HPRC), a first draft human pangenome reference has been generated (Liao *et al.* 2023). Parallel to this effort, several tools have emerged for graph construction (Li *et al.* 2020, Garrison *et al.* 2024, Hickey *et al.* 2024), read alignment on graphs (Li *et al.* 2020, Rautiainen and Marschall 2020, Sirén *et al.* 2021, Dabbaghie *et al.* 2023), variant discovery and genotyping (Garrison *et al.* 2018, Ebler *et al.* 2022, Prodanov *et al.* 2024, Söylev *et al.* 2024).

Computationally, two key components are central to most of these tools: representing a pangenome graph and the alignments to it. In this context, the most widely accepted formats are GFA (Graphical Fragment Assembly) for graphs and GAF (Graph Alignment Format) for alignments. Although a few tools have been developed to work with GFA files, there is a notable lack of tools designed to manipulate and analyze alignment files for pangenomes, comparable to SAMtools (Li *et al.* 2009), an essential tool in the linear reference genome domain.

Here, we present gaftools, a toolkit offering various features for GAF alignments, such as viewing, sorting, indexing, and realigning. It also preprocesses rGFAs, adding tags essential for viewing and sorting. We provide block gzip (bgzip)-compressed I/O support to improve space efficiency. gaftools is available as a PyPI package and on Bioconda (Grüning *et al.* 2018) and therefore easy to deploy.

## 2 Methods

In the following sections, we describe the main functionalities of gaftools and its different utilities.

### 2.1 Indexing and viewing of GAF files

The *gaftools view* command allows filtering a GAF file to alignments to specific nodes, paths, or regions. This also includes coordinate system conversion of alignment paths between “stable” and “unstable” (Li *et al.* 2020). The unstable system indexes each base by the node ID (a.k.a. segment id) and the offset on the node, whereas the stable system uses contig ID, independent of the graph nodes. Thus, by using *gaftools view --format,* conversion between the two coordinate systems is possible. As an example, a read from the NA12878 genome aligned to “>s249732>s326404>s249733” in the HPRC-r518 T2T-CHM13 Minigraph based on the unstable coordinate system can be converted to the same path expressed as “>chr19:18591208–18685851>GRCh38#0#chr19:18550488–18550808>chr19:18685851–18688039” in stable coordinates.

We also present a basic index structure for GAF files, which is essential for the view command to enable rapid access to alignments associated with user-specified nodes and regions. The index is an inverse dictionary, where the keys represent node IDs, and the values are lists of offsets within the GAF file indicating the locations where each node ID is found. Along with a bgzip-based compression of the GAF files, the offsets enable the view command to effectively traverse the GAF file and retrieve the desired alignments.

### 2.2 GFA to rGFA conversion

The reference GFA (rGFA) format is a strict subset of the GFA format used by Minigraph (Li *et al.* 2020), one of the three graph construction tools used by the HPRC (Liao *et al.* 2023) and has been proposed as a stable format for pangenome graphs.

gaftools subcommands such as *index*, *sort*, and *view* require an rGFA file as input. To enable the use of gaftools with standard GFA files—and to facilitate compatibility with other tools that may also require the rGFA format—we implemented the *gaftools gfa2rgfa* subcommand, which converts GFA files to rGFA. The implementation requires that the GFA file contains W lines, which encode the node traversal of the assemblies represented in the graph. The reference assembly, serving as the backbone of the graph, must be cycle-free. During conversion, the subcommand adds the following tags: SN (the name of the contig from which the node sequence originates), SO (the coordinate in the contig where the node sequence starts), SR (the order in which the contig was added to the graph), and LN (the length of the node sequence). While the LN tag is not required by the rGFA specification, we include it to facilitate rapid access to node lengths in downstream scripts that rely on this information.

### 2.3 GFA ordering and sorting of alignments

Efficient queries on large-scale datasets necessitate ordered and sorted data. However, in contrast to a linear reference, a general graph with cycles does not immediately imply a specific ordering. To address this, we propose an ordering and sorting approach specifically designed for pangenomes.

To order pangenome graphs (using *gaftools order_gfa*), we leverage the quasi-linear property of graphs incrementally built starting with a linear reference and then adding assemblies, e.g. using Minigraph (Li *et al.* 2020) (which produces rGFA) or Minigraph-Cactus (Hickey *et al.* 2024) (which can be converted to rGFA using *gaftools gfa2rgfa*). The presence of a linear reference path within the graph serves as a backbone for ordering and provides an orientation. We identify all the biconnected components that represent different variants in the graph, resulting in a continuous chain of these components for each chromosome. Here, we call these biconnected components “bubbles”. The articulation points of the graph here correspond to reference contigs, and we refer to them as “scaffold nodes”.

**Figure 1:**
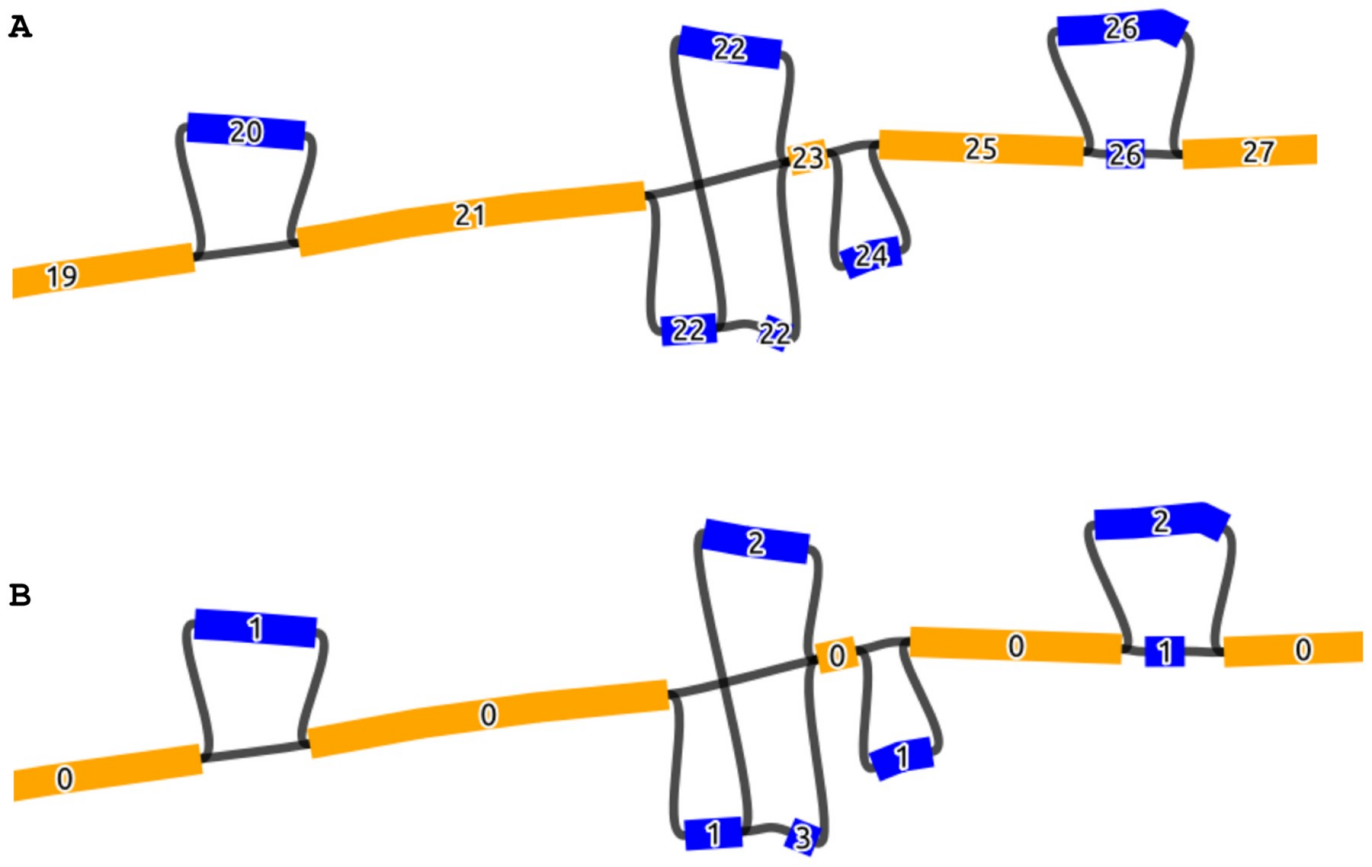
The Bandage figure (Wick *et al*. 2015) depicts the BO (A) and NO (B) tags. Blue nodes are bubble nodes and orange ones are scaffold nodes.

For this purpose, we introduce additional tags to the nodes of the graph, termed BO (Bubble Order) and NO (Node Order). The BO tags sequentially label scaffold nodes and bubbles detected in each connected component, where each component represents a chromosome. We begin at the scaffold node with the smallest reference coordinates (indicating the chromosome’s start). We then follow the detected bubbles, assigning the first scaffold node (the bubble source) a unique BO tag and an NO tag of 0 (all scaffold nodes get an NO tag of 0). All nodes within the bubble that follow this scaffold node are tagged with a single BO tag, incremented by 1 from the scaffold node’s BO tag. Subsequently, the scaffold node after the bubble gets a BO tag further incremented by 1. The NO tags inside the bubble sequentially label the nodes, starting from 1, based on the lexicographic order of the node IDs. Figure 1A shows a simple chain of 4 bubbles and 5 scaffold nodes. All nodes within a bubble are assigned the same BO tag. For the NO tag (Fig. 1B), scaffold nodes are assigned a value of 0, with subsequent nodes within the bubble incrementing by 1.

With this ordered graph structure, a sorted GAF file can be generated (*gaftools sort*) using the BO and NO tags as sorting keys in that order of precedence. This is necessary for various operations in order to optimize data processing and reduce redundant memory accesses.

### 2.4 Exact gap-affine realignment

Sequence-to-graph alignment is a challenging task and different tools come with different limitations. GraphAligner, for instance, is based on an edit distance model, which can produce biologically implausible gap placements. With *gaftools realign*, we allow for affine gap-cost based realignment of each alignment to its given path using the wavefront alignment algorithm (WFA) (Marco-Sola *et al.* 2021, 2023), which can be run in a multi-threaded mode to enhance performance.

**Figure 2:**
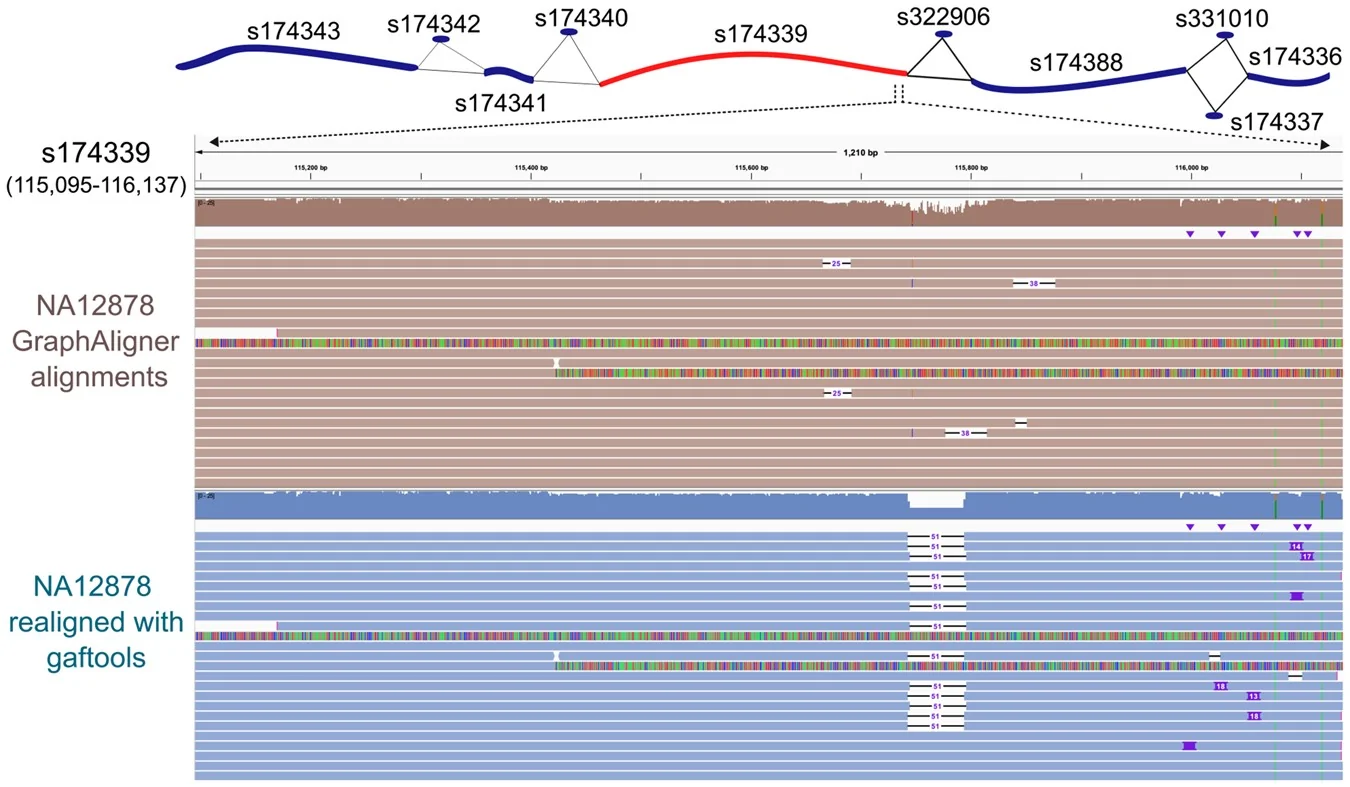
The IGV (Robinson *et al*. 2011) figure illustrates the ONT reads of NA12878 aligned to node s174339 in the HPRC-r518 T2T-CHM13 Minigraph pangenome graph using GraphAligner (depicted in brown), compared to the same alignments after being realigned with gaftools (depicted in blue). The pangenome path is displayed above and the red node is the source of the variant.

Figure 2 shows one such scenario where an alignment of NA12878 reads using GraphAligner to the HPRC pangenome obscures a deletion of 51bps but realignment of the same reads to the same positions enables the deletion to be captured.

### 2.5 Additional features

gaftools also provides additional functionalities, such as adding phasing information to alignments, determining the genomic path of a given node order, and generating statistics. First, phasing makes it possible to deduce which haplotype a read maps to in a given GAF file. Using *gaftools phase*, we append this information to the alignment file in the form of ps:Z and ht:Z tags, corresponding to phase set and haplotype, respectively. This data is based on *whatshap haplotag* TSV file output given as input (Martin *et al.* 2016). Additionally, we allow retrieving the genomic sequence corresponding to a specified GFA path (e.g., “>s106519>s106520<s488457>s106530>s106531”) using *gaftools find_path*, facilitating various downstream analyses. Furthermore, we provide comprehensive statistics for read alignments, including the number of primary and secondary alignments, aligned base counts, the number of reads with at least one alignment, mapping quality, sequence identity, and map ratio. In extended mode (using *--cigar* flag), we report additional information from the CIGAR string of the GAF file, including the total number of insertions, deletions, substitutions, and match regions.

## 3 Results

We assessed the runtime and memory consumption of each gaftools command using the graph alignments of NA12878 ONT (Oxford Nanopore Technologies) reads (∼14X depth of coverage) aligned to HPRC-r518 T2T-CHM13 using Minigraph (Table 2). Results show that gaftools is very fast and memory-efficient for all commands, with the exception of *realign*. The higher runtime and memory usage for *realign* are expected due to the computational demands of WFA alignment. In particular, our runtime when running on a single CPU core is comparable to GraphAligner when using 24 CPU cores, making *gaftools realign* a comparatively lightweight postprocessing step to tools like GraphAligner. We note that gaftools also supports multithreaded processing for *realign*, enabling even faster execution.

gaftools offers key functionalities not included in state-of-the-art tools such as VG (Garrison *et al.* 2018) and Minigraph/gfatools (Li *et al.* 2020, Li 2024) that work with GFA graphs and GAF alignments, and SAMtools (Li *et al.* 2009) that works with SAM/BAM alignments (alignments against a linear reference). Table 1 shows the shared and unique features, showing that gaftools offers functionalities previously only available for linear alignments. We would also like to note that for the stable/unstable conversion, Minigraph is able to produce alignments with either stable or unstable coordinates, however, the format needs to be specified before running the alignment, i.e. we would need to run the alignment step again if we wanted to change the format.

**Table 1:** The feature matrix outlines the functionalities of gaftools, alongside other tools offering similar capabilities.

	*Graph alignments*			*Linear alignments*
	gaftools	VG	Minigraph/gfatools	SAMtools
Conversion stable/unstable	✓	−	✓	N/A
Extract subset of alignments	✓	✓	−	✓
Alignment format supported	GAF	GAM, GAF	GAF	SAM, BAM, CRAM
Indexing alignments	✓	✓	−	✓
Graph ordering	✓	−	−	N/A
Alignment sorting	✓	✓	−	✓
Add haplotype tags	✓	−	−	−
(Re)align with affine gap costs	✓	−	−	−
Convert path to sequence	✓	−	−	N/A
Generate alignment statistics	✓	✓(GAM)	−	✓

**Table 2:** Runtime and memory consumption of gaftools functionalities using NA12878 alignments to HPRC-r518 T2T-CHM13 graph (with Minigraph) are given below.

Command	Runtime (h: mm)	Memory (GB)
view	<0:01	<1
view (–format)	0:20	2.2
index	0:01	2.2
order_gfa	0:01	2.2
sort	0:08	3.2
phase	0:05	1.8
realign	64:34	47
gfa2rgfa	09:03	29
find_path	<0:01	5.5
stat	0:04	1.7
stat (–cigar)	0:40	1.7

We performed the analysis using a single CPU core, with an Intel(R) Xeon(R) Gold 6136 CPU @ 3.00 GHz with 48 CPUs and 196 GB RAM machine.

## 4 Discussion

While the tool ecosystem to work with pangenomes is maturing, there are still considerable gaps compared to software infrastructure available to work with linear references. Here, we introduce gaftools as a versatile toolkit to support the practical use of pangenome references and provide missing functionalities. In particular, gaftools focuses on working with alignments of reads to pangenome reference graphs and offers a range of functionalities for the manipulation and analysis of GAFs, including viewing alignments, sorting, and indexing, which can be viewed as counterparts to common operations on linear alignments stored in SAM/BAM files. Beyond this, gaftools offers further functionalities like coordinate conversion between the stable and unstable coordinate systems, gap-affine realignment, and transferring of phase information into GAFs. gaftools has already proven valuable for the analysis for the ONT-1KG data set (Schloissnig *et al.* 2025), and we envision it will be instrumental for implementing future workflows for pangenome-based analyses.

## Data Availability

HPRC T2T-CHM13 pangenome graph can be found at https://zenodo.org/record/6983934. NA12878 GAF file can be found in https://ftp.1000genomes.ebi.ac.uk/vol1/ftp/data_collections/1KG_ONT_VIENNA/gaf/.
